# Price and distribution policies in healthcare marketing in Romania

**Published:** 2017

**Authors:** BI Coculescu, EC Coculescu, VL Purcărea

**Affiliations:** * “Titu Maiorescu” University, Faculty of Medicine, Bucharest, Romania; Center for Military Medical Scientific Research, Ministry of National Defence, Bucharest, Romania; ** “Carol Davila” University of Medicine and Pharmacy, Faculty of Dental Medicine, Bucharest, Romania; *** “Carol Davila” University of Medicine and Pharmacy, Faculty of Medicine, Bucharest, Romania

**Keywords:** price policy, distribution policy, marketing mix strategy, Romanian healthcare services

## Abstract

There is a principle similar to the theory of exchange in the marketing of health services, meaning that what is delivered to the target market (i.e. the beneficiaries) must be equal to or greater than what is to be received (i.e. the price). The price level in the marketing mix is influenced by how the consumer perceives the respective medical service and is quantified in the profit and the turnover of the organization respectively. The cost of the medical act as a whole is the value of all the tangible and intangible variables associated with it, and the planning, distribution and promotion of the product must be taken into account in the price setting.

## Introduction

The concept of marketing can be expressed in terms of four general groups of instruments known as “the four P’s of marketing”: 1. Product, 2. Price, 3. Placement, and 4. Promotion (McCarthy). The 4 P’s represent the seller’s point of view regarding the marketing tool that is available to influence the buyer and it can be translated into healthcare marketing by matching the terms: Service, Tariff, Access, and Promotion. The seller’s four P’s group corresponds to the client’s 4 C’s customer group (Lauterborn): 1. Customer requirements and wishes, 2. Cost to the customer, 3. Convenience and 4. Communication. What is important for the difference between consumer goods marketing and service marketing is the increase in the marketing mix, the central element of marketing strategy from “4 P’s” to “7 P’s”: Product, Price, Placement, Promotion, Physical evidence, Participants, and Process [**1**-**3**].

## Discussion

**Price policy**

The price of health products is the amount (cost) that the target market associates with the product offered by the medical organization. The price is one of the main elements with direct implications for all the activities and the marketing actions of the medical organization. It represents the exchange value of the health care service, meaning the amount of money that the patient offers to obtain that service in return for the benefits of using it [**2**-**4**].

Since patients are not well informed about the quality of health services in relation to the price of medical activity, the price strategy involves taking into account some factors that patients consider when addressing a specific service. These elements are also related to the patient’s involvement in the provision of the specific health service: the time spent by the patient as the the medical service is performed as a whole, the mental implications of the service for the patient (feeling of something new, fear, emotions, stress, etc.), physical effort the patient makes to obtain the health service.

For the purpose of positioning the global health product on the market, the application of a strategy of diversification of services will also be considered, that can be achieved by the medical organizations either by adding new features to the current offer or by employing better trained and better involved personnel than the competitor’s, or perform better image marketing.

The price policy is diversified according to the health services offered: prevention services, diagnostic and treatment services, medical recovery services, medical research services, etc.

**Distribution policy of healthcare services**

The distribution of health services is a complex process involving decisions regarding the place, time and methods of supplying the services. It encompasses all the activities that provide patients with access to healthcare services along with the relationship system established between providers, patients and other intermediaries. In the field of healthcare, distribution includes all the activities taking place in the space and the time separating the patient from the services provider [**5**,**6**].

The process of distribution of health services represents the actual materialization of the activity of medical organizations and, at the same time, it involves the accomplishment of the purpose of medical marketing, meaning the satisfaction of the patients’ health needs. The distribution of health services is ambivalent: it is performed at the patients’ request either by accessing the services within the medical institution (hospital, clinic, medical office, etc.) or by moving the provider of the medical services to the patient, in the case of beneficiaries requesting and requiring care at home. At the same time, an important factor that should be underlined is that there is a strong connection between the health services and their distribution system due to the influence that the medical service has on the patient.

The healthcare distribution network consists of all the locations in which the medical units (medical offices, clinics, diagnostic and treatment centers, hospitals, etc.) are found and, in which the provision of medical services to the patients is carried out.

An important role in the distribution strategy is that the health service provided is performed in the most pleasant and beneficial conditions for the target audience. Optimal distribution strategies, similar to those in the marketing of goods, should take into account several factors: location of the medical unit, time schedule, home medical facilities, comfort conditions, waiting periods, etc.

Establishing the patients’ needs and addressability of the target market is the starting point for an optimal distribution project. Knowing the patients’ expectations in choosing the most effective way to distribute the medical services should take into account the possibility of patients being satisfied by the services provided.

Cost-effectiveness should be geared to creating spaces as close as possible to the patients’ needs, to fit within the widest possible range of working hours, and to make access to healthcare as easy and convenient as possible compared to other organizations in the field. The easier these elements are to achieve and, consequently, to be obtained by the patients, the greater their interest in obtaining these services.

## Conclusions

When determining the correct price level, healthcare organizations should take into account the way patients appreciate the value of health services and other factors that influence the direction of a particular medical service. The value of a service is defined by patients in a number of ways: “value is a low price” or “value is what I get for the price I pay” or “value is what I want from a service”. The patient will no longer resort to a particular healthcare service if he/ she negatively perceives the value of that service, and that happens when the price paid is higher than the benefit he/ she obtains. The net benefit of the service, which is the difference between the amount of benefits received and the sum of the costs associated with that service is taken into account in determining the value of the health service by the patients. A wrong perception of the value of medical services can be influenced by the erroneous interpretation of how patients define the value of that service or by suggesting a lower quality of the service due to uncompetitive prices.

Although strong medical organizations can charge higher prices for medical services, the price supplement cannot be exaggerated. Unless appropriate investments are made in the value of the service, price differences will make the medical organization more vulnerable to the competition, from those with lower-priced medical services. Customers/ patients may be willing to change their healthcare provider if they can no longer justify to themselves that a more expensive service is worth the money [**1**,**3**].

In terms of potentiating the correlation with the other components of the marketing mix, the distribution of strategy also targets the selection of intermediary partners and those involved in the overall medical policy of the organization. The choice of a particular strategy must be the result of a profound analysis aimed at balancing and efficiently coordinating all the activities required for the optimal deployment of the healthcare distribution phase.

Therefore, the entire process of establishing the distribution strategy involves the following conditions:

• identifying the needs of the patients at the target market level, related to the level of distribution services;

• defining the objectives of the distribution strategy;

• developing the types of distribution strategies;

• analysis of selection criteria (social, economic, control);

• choosing the best strategic distribution option.

Since distribution is a dynamic component of the marketing mix, the distribution strategy needs to be reformulated and adjusted periodically, depending on the change in the organization’s goals, the phase the organization is going through in its evolution, changes in the target market, and the emergence of more efficient forms of distribution [**5**,**7**]. By implementing its distribution strategy, the medical organization must seek to harness the full potential of distribution as a component of the marketing mix, in conjunction with its other elements.
